# Corrigendum

**DOI:** 10.1002/ehf2.13858

**Published:** 2022-02-20

In the paper by Obradovic *et al*.,^1^ the first name and surname of the authors have been interchanged.

The correct names of the author are listed below:

Slobodan Obradovic, Boris Dzudovic, Bojana Subotic, Jovan Matijasevic, Zorica Mladenovic, Aleksandar Bokan, Jadranka Trobok, Sandra Pekovic, Sonja Salinger‐Martinovic, Ljiljana Jovanovic, Ljiljana Kos, Tamara Kovacevic‐Preradovic, Maja Nikolic, Vladimir Miloradovic, Ana Kovacevic‐Kuzmanovic, Zec Nenad, Natasa Markovic‐Nikolic, Ilija Srdanovic, Zoran Gluvic, Srdjan Kafedzic, Sasa Pancevacki, Aleksandar Neskovic and Stavros Konstantinides.
